# *Listeria monocytogenes* Endophthalmitis—Good Outcome With Rapid Diagnosis Using MALDI-TOF and Treatment With Benzylpenicillin and Trimethoprim–Sulfamethoxazole

**DOI:** 10.1155/crop/6380811

**Published:** 2025-05-28

**Authors:** Thomas Ledger, Martin Plymoth, Mark Douglas

**Affiliations:** ^1^Centre for Infectious Diseases and Microbiology, Sydney Infectious Diseases Institute, The University of Sydney at Westmead Hospital, Sydney, New South Wales, Australia; ^2^Western Sydney Local Health District, Sydney, New South Wales, Australia; ^3^Department of Clinical Microbiology, Umeå University, Umea, Sweden; ^4^Storr Liver Centre, The Westmead Institute for Medical Research and The University of Sydney, Sydney, New South Wales, Australia

**Keywords:** endophthalmitis, *Listeria monocytogenes*, matrix-assisted laser desorption ionization time of flight mass spectrometry

## Abstract

**Purpose:** Describe a case of rapidly diagnosed *Listeria monocytogenes* endophthalmitis, with a clearly established route of endogenous seeding, treated with dual antibiotics, with outcomes better than many reported.

**Observations:** A 79-year-old male developed *Listeria monocytogenes* endophthalmitis after a gastrointestinal infection with an associated bacteraemia. Rapid microbiologic diagnosis was obtained via matrix assisted laser desorption ionization–time of flight mass spectrometry (MALDI-TOF). The case was managed with 3 weeks of benzylpenicillin and 6 weeks of trimethoprim–sulfamethoxazole. Visual acuity in the affected eye was preserved with a mild to moderate residual deficit.

**Conclusion:** Clinicians should be aware of the potential for *Listeria monocytogenes* endophthalmitis associated with preceding gastrointestinal symptoms. Better outcomes are associated with rapid diagnosis, and the use of dual antimicrobial therapy should be considered.

## 1. Case

A 79-year-old male from regional New South Wales, Australia, presented to a local emergency department with confusion, 1 day of fevers and rigors, and 3 days of vomiting and diarrhea. He had multiple medical issues including Stage 3a chronic kidney disease, Type 2 diabetes mellitus on oral hypoglycaemics, transcatheter aortic valve replacement 6 years prior for severe aortic stenosis, a previous cerebrovascular accident without ongoing sequelae, hypertension, benign prostatic hypertrophy, depression, and cataract surgery. He was an active smoker with a 70-pack-year history and reported consuming around nine standard units of ethanol on weekends. He lived independently at home with his wife.

On presentation, his temperature was 38.2°C, blood pressure was 126/71 mm Hg, and heart rate was 84 beats per minute. There was no clinical meningism. Acute urinary retention was present, and a urinary catheter was inserted. Investigations showed an elevated C-reactive protein (CRP) (50 mg/L (normal range < 10 mg/L)), an elevated white blood cell count (8.7 × 10^9^/L), and a mild acute kidney injury with an estimated glomerular filtration rate (eGFR) of 30 mL/min. No acute intracranial abnormalities were seen on a computed tomography brain scan.

The patient was admitted for supportive management of gastroenteritis, with intravenous fluids given. One of two blood cultures flagged positive for Gram-positive rods, in an anaerobic bottle (Bactec Lytic, Becton, Dickinson and Company, Canada), which were identified the following day as *Listeria monocytogenes* by matrix-assisted laser desorption ionization–time-of-flight mass spectrometry (MALDI-TOF). Directed intravenous benzylpenicillin was commenced, at a renally-adjusted dose of 1.8 g every 4 h, and a single dose of gentamicin 240 mg was given. Lumbar puncture revealed elevated cerebrospinal fluid (CSF) protein levels (0.95 g/L (normal, < 0.69 g/L)), normal CSF glucose (4.4 mmol/L (normal, 2.5–4.5 mmol/L)), and cell count (5 leukocytes × 10^6^/L, which corrected for erythrocytes of 2500 × 10^6^/L, was within normal limits). An in-house multiplex CSF PCR panel was negative for *L. monocytogenes, E.coli* K1, *H. Influenzae*, *N. meningitidis*, *S. pneumoniae*, *S. agalactiae*, cytomegalovirus, enterovirus, herpes simplex virus (HSV-1 and HSV-2), human parechovirus, human herpes virus-6, varicella zoster virus, and *Cryptococcus neoformans/gattii*). Collateral history from family suggested several potential sources of *L. monocytogenes* infection, including ingestion of brawn (pig-head cheese) from a local delicatessen 2 weeks prior to symptom onset, sliced rock melon 2 weeks prior, watermelon 4 weeks prior, and peaches from a local orchard 4 weeks prior.

**Figure 1:**
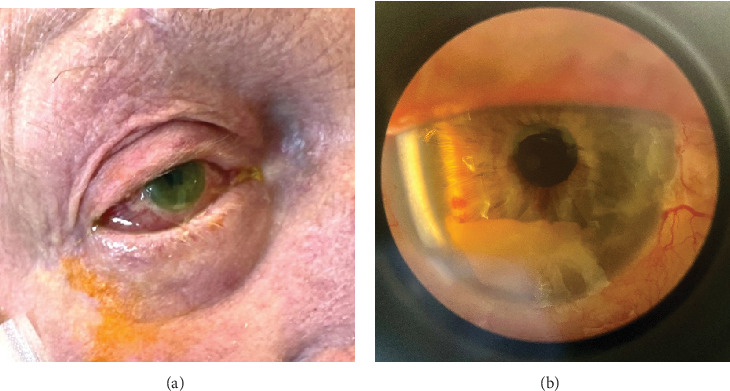
Evolving right eye *L. monocytogenes* endophthalmitis shown at (a) Day 3 of presentation with conjunctival hyperaemia, chemosis, and formation of hypopyon and (b) Day 14 (Day 10 postintravitreal antibiotics) through slit lamp examination with evolving hypopyon (2.8 mm) and ongoing conjunctival injection.

On the second day after presentation, the patient reported mild right-sided eye pain, with blurred vision by the third day. Initial examination revealed conjunctival hyperaemia of the right eye, with chemosis, sluggish pupillary response to light and cloudiness in the anterior chamber by the third day (Figure 1a). Right intraocular pressure was raised at 31 mm Hg, and visual acuity was less than 6/60. Brimonidine 0.2%, dexamethasone 0.1%, ofloxacin 0.3%, latanoprost 0.005%, and timolol 0.5% eye drops were commenced for endophthalmitis after ophthalmological review.

The patient was transferred to a tertiary hospital. On arrival, visual acuity was to light only and eye pain had resolved. A comprehensive ophthalmological exam demonstrated a right hypopyon (Figure 1b). There was no visibility of the posterior chamber. B-scan ultrasonography showed vitritis near the retinal surface, with a flat retina. A vitreal tap aspirated 0.4 mL of fluid and injected of 0.1 mL (2.25 mg) ceftazidime, 0.1 mL (1 mg) vancomycin, and 0.1 mL (0.4 mg) dexamethasone. Visual acuity improved to hand movements posttap. High-dose oral trimethoprim–sulfamethoxazole (320/1600 mg twice daily) was added for meningeal penetration [1]. Given the patient's high anesthetic risk and poor visual prognosis, he was deemed unsuitable for surgical intervention. Visualization of the posterior chamber improved gradually, with vitreal clearing by Day 9. Posterior synechiae formation was seen, and atropine eye drops were commenced. By Day 11, intraocular pressures had normalized. Visual acuity was finger counting at 30 cm and remained so during the rest of the admission. Gastroenteritis symptoms had resolved.

*L. monocytogenes* was isolated from intravitreal, blood, and stool cultures and was susceptible to penicillin (minimum inhibitory concentration (MIC) 0.50, Etest, bioMérieux) and trimethoprim–sulfamethoxazole (MIC 0.047, Etest, bioMérieux). Whole genome sequencing (Illumina Technologies) identified the isolate as *L. monocytogenes* sequence Type 1. Transthoracic echocardiography showed a well-seated bioprosthetic aortic valve without evidence of endocarditis.

A peripherally inserted central line was placed, and the patient was discharged to a hospital in the home program, receiving a total of 3 weeks of intravenous benzylpenicillin, given as 10.8 g daily by continuous infusion after discharge, and a total of 6 weeks of trimethoprim–sulfamethoxazole. On follow-up after the course of antibiotics, visual acuity had improved to 6/18, classified as mild to moderate impairment by the World Health Organization.

## 2. Discussion

Classically described as a cause of food-borne gastroenteritis, with risk factors for invasive disease being age extremes, pregnancy, and significant immunosuppression [2], *L. monocytogenes* as a cause of endophthalmitis is rarely reported. This is only the second report of listeria endophthalmitis in Australia in a 40-year period, and only the fourth outside of the EU and United States [3, 4]. There has been an increase in *L. monocytogenes* endophthalmitis cases reported over time, likely due to an increase in case identification due to availability of testing and clinician awareness, changes in data collection and reporting, dietary intake, and increased availability of publishing venues [4].

*L. monocytogenes* is a motile aerobic Gram-positive rod that can replicate in a wide variety of food groups and across a broad range of salinity, acidity, and temperatures, including when refrigerated (−1.5° to 45°) [2, 5]. It has been found in soil in multiple biomes and asymptomatically in livestock feces [6], but the lack of clearly documented acquisition routes outside of food-related outbreaks of gastroenteritis means it is considered a foodborne illness [4].

After ingestion, *L. monocytogenes* traverses the gastrointestinal barrier and disseminates to affected body sites via blood and lymph. After receptor-mediated endocytosis on gastrointestinal epithelial cells, it is able to ex-vacuolate and replicate within the intracellular environment [2]. Virulence factors which facilitate vacuole escape include LIPI 1 and 2, which are coded by the Pathogenicity Island 1 [2], and were identified in two cases of *L. monocytogenes* endophthalmitis that underwent metagenomic testing [7].

Foods commonly associated with *L. monocytogenes* transmission include dairy products, meats (such as ham, salami, and, in this case, brawn), seafoods, cheeses and melons [8]. The median incubation time from exposure to onset of symptoms is approximately 10 days, based on a series of 48 cases with defined exposures [9]. However, it is likely the incubation time varies according to inoculum dose, host immunity, and virulence factors [9].

*L. monocytogenes* was first described in 1926 in an outbreak of gastroenteritis among rabbits and guinea pigs [2]. The potential for *L. monocytogenes* to cause keratoconjunctivitis was first demonstrated by direct inoculation in 1934 [10], and the first case of endophthalmitis in humans was reported in 1967 [11]. Although more common in animals exposed to contaminated fodder, *L. monocytogenes* is a rare cause of endophthalmitis in humans [10]. In a recent systematic review, 43 cases of *L. monocytogenes* endophthalmitis were identified [4]. The majority of cases of endophthalmitis are endogenous, that is, via haematogenous spread, with only four reported exogenous cases (following eye trauma, refractive surgery, and insertion of corneal lenses) [4]. In the systematic review, the median age was 61 years, with half of the patients having identified immunosuppression, broadly defined to include diabetes, cancer, and past corticosteroid use. Fifty-seven percent of cases were in men. A hypopyon was reported in 60% of cases (*n* = 26), with hypopyon pigmentation associated with a better prognosis (8/8 improving visually vs. 11/17 without) [4].

This case is distinguished from previous cases by the preceding gastroenteritis, the three positive cultures (stool, blood and vitreal) clearly defining a route of spread to the eye, and the use of MALDI-TOF to expedite diagnosis. In comparison, most reported cases presented with predominantly ocular symptoms, and the majority of positive cultures were from the eye (31 of 35 reported cases with cultures), rather than blood (only 3 of 14 reported) [4]. In a French registry of *L. monocytogenes* bacteraemia and neurolisteriosis, only 22% and 15% of cases, respectively, had associated diarrhea [12].

Our patient was treated with systemic benzylpenicillin and trimethoprim–sulfamethoxazole and received an initial intravitreal dose of ceftazidime, dexamethasone, and vancomycin [11, 13]. There is a wide variety of reported prescribing patterns for both *L. monocytogenes* and endogenous endophthalmitis [4, 14]; in the systematic review, no two cases received the same regimen. Ampicillin or amoxicillin was the most commonly prescribed beta lactam (22/35), with most cases receiving additional vancomycin (17/33) or gentamicin (15/33). Three quarters (24/33) received intravitreal antibiotics [4]. For the reported patient, infused benzylpenicillin was able to facilitate a discharge home via our hospital in the home program, and infusers are not locally available for amoxicillin and ampicillin. We added trimethoprim–sulfamethoxazole as an oral agent with meningeal penetration, with in vivo reports of improved outcomes with dual use of ampicillin and trimethoprim–sulfamethoxazole [15], and to avoid the risks of ototoxicity and need for ongoing intravenous access associated with gentamicin [16]. We chose to discontinue further intravitreal injections based on literature supporting adequate intravitreal concentrations of parenteral antibiotics in the context of endogenous endophthalmitis, possibly due to changes in the porosity of the blood-eye barrier associated with systemic infection [11, 13].

Many cases had received steroids prior to the identification of *L. monocytogenes* endophthalmitis, including in 8 of 16 cases without a background history of immunosuppression [4]. It is hypothesized that this is because of the primarily ocular presentations of *L. monocytogenes* endophthalmitis, and a more common differential diagnosis of red eye is inflammatory uveitis [11]. Antibiotic duration varied from 5 to 60 days, with a median duration of 21 days [4]. Outcomes were generally poor overall, with 39% of cases remaining blind, and only one third reporting a return to baseline vision [4].

Antimicrobial resistance in *L. monocytogenes* is reported and differs markedly by geography [10] [17]. The isolate we report was susceptible to all tested antibiotics, including penicillin and trimethoprim–sulfamethoxazole. This is consistent with a recent analysis of 100 food chain isolates in Australia that reported low levels of resistance to ciprofloxacin (2%) and erythromycin (1%); all specimens were penicillin susceptible [17]. Worldwide rates of resistance differ markedly, ranging from 0% to 100% penicillin resistance and 0%–17.8% ciprofloxacin resistance [17].

The culprit food in this case, as in many reports, was hard to identify [9]. Outside an outbreak with a cluster of cases defined by ingestion and geography, the dietary history reported is often vague, and timelines are unclear. It is not local practice to perform exhaustive microbiological sampling of potentially affected foods outside of these circumstances.

In summary, we report a rapidly diagnosed case of *L. monocytogenes* endophthalmitis presenting with a prodromal gastrointestinal illness in the absence of immunosuppression, diagnosed by MALDI-TOF, with a rapid diagnostic workup following the report of visual changes. We describe a good visual outcome after treatment with dual benzylpenicillin and trimethoprim–sulfamethoxazole.

## Data Availability

The data that support the findings of this study are available on request from the corresponding author. The data are not publicly available due to privacy or ethical restrictions.
